# Culturally and contextually adaptive indicators of organizational success: Nunavik, Quebec

**DOI:** 10.17269/s41997-022-00704-x

**Published:** 2022-11-30

**Authors:** Sarah Louise Fraser, Marie-Claude Lyonnais, Mylene Riva, Christopher Fletcher, Nancy Beauregard, Jennifer Thompson, Raymond Mickpegak, Laury-Ann Bouchard

**Affiliations:** 1 Centre de Recherche en Santé Publique (CReSP), University of Montreal and CIUSSS du centre Sud-de-l’Île de Montréal, Montreal, Quebec Canada; 2 School of Psychoeducation, University of Montreal, Montreal, Quebec Canada; 3 Centre de Recherche CHU de Québec, Université Laval, Quebec City, Quebec Canada; 4 Department of Geography, McGill University, Montreal, Quebec Canada; 5 École de Relations Industrielles, Université de Montréal, Montreal, Quebec Canada; 6 Makivik Corporation, Montreal, Quebec Canada

**Keywords:** Program evaluation, Community health, Organizational practices, Indigenous health, Évaluation de programme, santé communautaire, pratiques organisationnelles, santé des Autochtones

## Abstract

**Objective:**

This study aimed to develop a preliminary guide to culturally and contextually relevant indicators to assess community resources in the 14 communities of the Inuit territory of Nunavik, Quebec.

**Methods:**

As part of the Community Component of Qanuilirpitaa? of the 2017 Nunavik Health Survey, data were collected from 354 organizations located across Nunavik. Data were collected via short structured interviews with representatives of the organization. An inductive qualitative analysis was conducted to identify emerging themes describing the contexts that influence organizations, how key informants conceptualized what is a successful resource, and the facilitators and needs to achieving these indicators of success. Inuit partners were involved throughout the project to offer insight and to ascertain its pertinence and validity.

**Results:**

Interviews revealed structural and community realities that influenced organizations. Three main indicators were used to describe successes: (1) team efficiency and dynamics; (2) accessibility of the resource; and (3) ability to impact clients and the community. The third indicator was by far the most discussed indicator of success. Participants and leaders offer suggestions as to how to achieve these indicators and advocate for the conditions necessary for organizational sustainability.

**Conclusion:**

This data-driven framework suggests that the measures of success that are frequently used by funding agencies (e.g., number of people reached, number of activities) may not fully represent the potential of local services in a given community. Indeed, services may be creating job opportunities for Inuit, instilling pride, offering cultural opportunities, and increasing capital (human, economic, health) within the community, all of which are equally important indicators of success that may more adequately further improve the social determinants of health among communities.

## Résumé

**Objectif:**

Cette étude visait à élaborer un guide préliminaire d’indicateurs culturellement et contextuellement pertinents pour évaluer les ressources communautaires dans les 14 communautés du territoire inuit du Nunavik, au Québec.

**Méthodes:**

Dans le cadre du volet communautaire de Qanuilirpitaa? de l’Enquête sur la santé au Nunavik de 2017, des données ont été recueillies auprès de 354 organisations situées dans tout le Nunavik. Les données ont été collectées via des entretiens courts et structurés avec des représentants de l’organisation. Une analyse qualitative inductive a été menée pour identifier les thèmes émergents décrivant les contextes qui influencent les organisations, la façon dont les informateurs clés ont conceptualisé ce qu’est une ressource réussie, ainsi que les facilitateurs et les besoins pour atteindre ces indicateurs de réussite. Des partenaires inuits ont été impliqués tout au long du projet afin d’offrir leur point de vue et d’en vérifier la pertinence et la validité.

**Résultats:**

Les entrevues ont révélé des réalités structurelles et communautaires qui ont influencé les organisations. Trois indicateurs principaux ont été utilisés pour décrire les réussites : 1) l’efficacité et la dynamique de l’équipe; 2) l’accessibilité de la ressource; et 3) la capacité d’avoir un impact sur les clients et la communauté. Le troisième indicateur était de loin le plus discuté des indicateurs de succès. Les participants et les dirigeants offrent des suggestions sur la façon d’atteindre ces indicateurs et plaident en faveur des conditions nécessaires à la durabilité organisationnelle.

**Conclusion:**

Ce cadre axé sur les données suggère que les mesures de réussite fréquemment utilisées par les organismes de financement (par exemple, le nombre de personnes atteintes, le nombre d’activités) peuvent ne pas représenter pleinement le potentiel des services locaux dans une communauté donnée. En effet, les services peuvent créer des possibilités d’emploi pour les Inuit, susciter la fierté, offrir des possibilités culturelles et accroître le capital (humain, économique, santé) au sein de la communauté, autant d’indicateurs de réussite tout aussi importants qui peuvent contribuer de façon plus adéquate à améliorer les déterminants sociaux de la santé au sein des communautés.

## Introduction

Community services such as grocery stores, community centres, libraries, and schools are social determinants of health, meaning that they are resources that are necessary for health and well-being of the populations served (Reading & Wien, 2009). There is widespread evidence that Inuit of Canada are underserved on all social determinants of health (Inuit Tapiriit Kanatami, 2014; Unicef, 2009). There has been a call for developing culturally adapted and safe services for Indigenous people, and more specifically for the region of Nunavik, with the intent of addressing the social determinants of health, including better access to health and social services, community services, affordable food, and more (Breton, 2021; Inuit Tapiriit Kanatami, 2014; Pernet, 2018). With these calls for action, funding has been made more readily available for communities and organizations to develop programs and services. However, along with this funding, funders request regular reports and annual evaluations of services. One of the challenges with evaluation reporting is that existing criteria can be incompatible and even sometimes detrimental to the community initiatives because the accepted or expected criteria reflect different sets of cultural norms (Dettlaff & Fong, 2011; Gill et al., 2016). Indeed, evaluating programs and services in cultural minority groups has been recognized as a challenge for decades (Aktan, 1999; Ginsberg, 1988; Griner & Smith, 2006; Lee, 2007). In the context of Northern Canada, a consistent priority shared by Inuit is the development of culturally safe evaluation criteria to ensure that evaluations are based on contextually relevant information and done in culturally safe ways (Breton, 2021; Inuit Tapiriit Kanatami, 2014; Pernet, 2018; St-Pierre, 2021). In this article, we develop a preliminary outline of contextually relevant indicators of success based on the self-evaluation of service providers in the region of Nunavik. Nunavik is the northern region of the province of Quebec, and one of the four large Inuit territories in Canada.

### Evaluating services for underserved groups: paradoxes and opportunities

There are at least two broad schools or approaches to evaluation. In the first, evaluation aims to rigorously document quantitative measures to justify the use of limited public resources (Fitzpatrick et al., 2004; Luke et al., 2013). Organizations might develop standardized procedures and frequent audits to document efficiency, productivity, and cost-effectiveness. When government or private moneys are used to fund these organizations, evaluation is generally requested as a form of accountability to boards of directors and to the general public (Fitzpatrick et al., 2004). This mode of evaluation has been criticized as limiting because it cannot account for the complex ways that services intersect with social and cultural systems within which they unfold (Chouinard, 2016; Rossetti & Wall, 2017). This gap can be seen in colonized societies where colonial structures have explicitly and implicitly imposed both universal sets of evaluation criteria as well as standardized approaches in order for evaluations to be viewed as normatively valid (Chilisa et al., 2016; Chouinard, 2016; Kirmayer, 2012). Critical thinkers suggest that these normative approaches rely on markers of success that have been favoured by capitalist market–driven societies (Blais, 2020; Cloos, 2011). Such criteria may favour forms of efficiency and economic sustainability in health services, notably by focusing on indicators such as the number of people reached, the number of sessions attended, or changes in clients’ symptoms or behaviours (Chen, 2005). These evaluation criteria can then become ingrained within individual and group psyche as being *the* benchmark of success (Quijano, 2007) through a variety of mechanisms including policy and funding. For instance, government organizations and other funding agencies will rely on these criteria to choose which services to fund and how much funding they receive (Duignan, 2002). From a critical standpoint, relying solely on evaluative criteria established by external non-community members may offer a fragmented appreciation of the actual impact that services may have on the overall well-being of a community. Evaluations conducted with predefined and contextually irrelevant criteria can replicate dynamics of surveillance and control of minority groups. Evaluators can participate in shedding light on social inequalities by adapting their approaches and using their evaluation tools to do so (Thomas et al., 2018). Furthermore, these approaches may inadvertently steer organizations in directions that contradict their actual mandates. For example, if an organization is meant to serve underprivileged and hard-to-reach individuals within a community but then evaluates its services using the criteria “number of people reached,” it may end up prioritizing services for “easier-to-reach” individuals to meet the expected quantitative markers.

To address this problem, many researchers and activists working with underserved minorities advocate for more critical and social justice–oriented approaches. Many frameworks have been developed to support these forms of evaluation including transformative evaluation, feminist evaluation, Gender Environment and Marginalized voices framework, empowerment evaluations, critical systems evaluations, and equity-focused evaluations, to name a few (Brisolara, 2014; Fetterman & Wandersman, 2005; Mertens, 1999; Reynolds, 2014, Stephens et al., 2018). Many authors have offered guidelines to culturally adapt the evaluation of programs and services in different socio-cultural contexts (Barrera Jr et al., 2011; Casillas & Trochim, 2015; Centers for Disease Control and Prevention, 2014; Duignan et al., 2003). For instance, Hood et al. (2015) developed a contextually responsive framework that ensures that researchers do not interpret people’s everyday realities as “cultural specificities” when in fact they may be structural inequalities that influence everyday lives. In sum, these frameworks all aim to learn from and help adapt the services based on the needs that are observed through the evaluations. Hence, if organizations are experiencing the realities of structural inequalities such as lower funding for certain populations, or difficulty establishing trusting relationships with certain organizations due to systemic racism, then accounting for these dynamics in evaluations allows organizations to make explicit, learn from, and work towards addressing the root causes of these problems. Contextually responsive evaluations can thus be more empowering and transformative for the organizations as well as for the communities served, as they are imbricated within the offer of services. The design of the evaluation criteria and process should be developed in participation with those for whom the services are meant to serve and must be anchored in the eco-systemic realities of the given population (Al Hudib et al., 2016; Chouinard, 2016; Cotton et al., 2022; Stanton, 2014). Many articles offer guidelines that accompany evaluators throughout the evaluation process. However, very little is written about what criteria may be used to assess programs and organizations among underserved populations. Criteria building is only one of many steps in the evaluation process. However, by redefining success in ways that are contextually and culturally relevant, services can reorient their approaches, their missions, and their planning, in ways that assess the everyday barriers faced by service providers and beneficiaries of the services. Then, once the organization has developed a functioning that is adapted to the realities of the community, the services become more accessible and used by the population the organization is meant to serve, ultimately improving community health and resilience (Laliberté et al., 2012; Reading & Wien, 2009).

Indigenous communities in Canada have had the immense task of developing new organizations and transforming existing services in colonial realities. We uphold that the objective of any evaluation of Indigenous services must be driven by the Indigenous peoples with the intent of supporting the transformation of the organization and its services, if and only if the evaluation is seen as a useful process for the functioning of the organization. We also posit that all organizations, no matter their location or mandate, have a life of their own and are in constant adaptation and transformation. It is in this spirit that the following project was conducted. The objective was to identify the criteria that staff working for organizations throughout the region of Nunavik use to describe the functioning of their organization in a positive light. These self-defined criteria then allow other organizations to reflect on the benchmarks that they may wish to use for their own organizational functioning, all the while considering the barriers and strengths they experience as organizations serving underserved Inuit in Northern Canada.

## Methods

The data were collected as part of a larger study through the Nunavik Regional Board of Health and Social Services (NRBHSS), the *Qanuilirpitaa? 2017* survey (hereafter Q2017), about the state of health of the Nunavik population. Q2017 (meaning “how are we now?”) was conducted for and by Inuit on board the Amundsen, an icebreaker repurposed for science, which travelled to the 14 communities of Nunavik in 2017 and 2018 (see Hamel et al., 2020). While there were several components of the Q2017 to evaluate physical and mental health in Nunavik, the data reported here pertain specifically to the community component of the survey. To document community well-being, Fletcher et al. (2022) developed the IQI^1^ model of community health and well-being as well as a contextually relevant framework for social determinants of health.

The data analyzed for the current study were collected in two phases. In the first phase in 2017, non-Inuit research assistants began to conduct brief interviews by phone over a 2-month period to start identifying key organizations in each of the communities, and the services they offer. Organizations were defined broadly based on Latham and Locke’s (1991) definition that suggests that organizations are rational entities with a goal. This includes any established organization, group of key people offering a service, or community initiative offering support, goods, or programs to the community. The research assistants then travelled on board the Amundsen with Inuit partners and spent between 1 and 7 days going door to door in each community to identify, characterize, and map out all the services within that community. Time constraints imposed by the sailing schedule of the Amundsen meant that the time spent in some communities was sometimes limited. Therefore, the research assistants returned to eight of the communities between January and March of 2018 to interview the representatives of services that had been missed in the first phase. They worked with an Inuit research assistant hired to work on various connected research projects, including this one.

Through this process, the research identified 356 organizations throughout Nunavik. In a second phase, semi-structured interviews of 10- to 60-min duration were conducted with key informants from each organization. Key informants were asked to describe the functioning of the services offered, including the number of employees, the types of services and programs offered, the target clientele for each program, the ways in which culture was being reinforced in the services, the barriers to use of the specific programs, the successes and strengths of the organization or of specific programs that their organization offered, any challenges they experienced as an organization, and what they felt was missing for their community (for more details regarding methodology, see Fletcher et al., 2021). This rich qualitative set of data allowed a team of non-Indigenous academic researchers and Inuit community leaders to explore culturally and contextually adaptive evaluation criteria. The first author is a researcher who has been active in community-based action research in the region for over 10 years and has developed strong relationships with community members in various communities in the region. Exchanges with community leaders then allowed the team to iteratively construct an evaluative framework based on Inuit perspectives of what success looks like within community services as well as the challenges and facilitators that impact the success of programs.

## Analysis

Each interview was audio-recorded and transcribed. Participant narratives that pertained to the themes of cultural reinforcement, development over time, and successes, strengths, and challenges were coded according to three broad categories: (1) the contextual realities that impact organizations, (2) the ways in which people speak of success within their organization, (3) the facilitators and needs to achieve success within an organization. These three categories allow us to reflect on the structural and systemic realities that influence organizations, and document how they impact the creation of indicators of success, which may, in the field of organizational evaluation be understood as “performance”. It is important to note here that success (or performance) are not defined a priori and emerge from the narratives of key informants as they describe what they feel pleased about, and what they hope for regarding the organization they work for. Two members of the research team initially coded the transcripts from three of the 14 communities to establish a coherent, rigorous approach to coding. Each researcher then started to organize themes individually. They met regularly to discuss similarities and differences in understandings, came to an agreement, and then returned to the data to explore the relationships between the various findings, as well as how to illustrate these relationships visually in a diagram. The data pertaining to the remaining communities were then coded in constant reference to the diagram, which was adapted iteratively and as necessary to ensure its correspondence with the findings based on ongoing exchanges with two community leaders. As the contexts, indicators of success, and the facilitators and needs were being identified, the two first authors explored how they were inter-related. Nvivo software was used for data analysis. Lennie’s (2006) criteria of trustworthiness for participatory evaluations were used to critically assess our own data collection and analysis process.

## Results

### Description of resources

Overall, a total of 356 organizations were identified across the 14 communities of Nunavik. There were between 19 and 43 organizations per community with smaller communities having fewer than larger communities. Here, we consider “small” communities to range between 200 and 900 inhabitants, and “large” communities to range between 1400 and 3000 inhabitants. The nature of the organizations included health-related services such as health and social programs offered at community clinics and youth protection services as well as more general community services such as municipal services, churches, and police and fire departments, and gas stations, local radio stations, and sewing centres. Each service offered a range of activities and programs, from community kitchens and *kamik*-making workshops (Inuit sealskin boots) to elder field trips and circus initiation classes. Most community-led resources were run by local Inuit community members, and included the gas station, local co-op (grocery), municipal services, and sewing centre. Institutional organizations funded by the provincial government were run primarily by non-Inuit staff. These services included schools, youth protection services, health clinics, and social services.

To build an adaptive framework, we organized the findings in three sections: (1) the contextual realities that influence organizations, (2) the indicators of success as described by organizational members, and (3) the organizational facilitators and needs to achieving this success.

### Contextual realities

Two broad contextual realities played an important role in how organizations discussed the function and successes of their services: colonization and its impact on communities, and geographical realities.

To begin, *the ongoing effects of colonization* were a prominent theme when discussing the community realities that impact organizations. Colonization was described as having heavily influenced community and individual well-being, trust that community members hold towards services, and the cultural threads that make individuals and groups feel whole. Inuit and non-Inuit participants focused on different perspectives on the relationship between colonization and health. The colonial legacy impacts the health and well-being of people suffering from transgenerational trauma. Individuals with more severe mental health challenges and addictions may have difficulty using any available service. Many people are also simply reluctant to consult services because of shame, or because they do not trust services provided by non-Inuit. Participants also described colonization as a factor that influences the personal health and well-being of clienteles as well as the confidence they have in themselves to participate in services and create change for themselves and for others. Organizations felt that the clienteles that they served had many different and complex needs with multiple social determinants of health not being met. As an example, a service might feel that a community member needs food, housing, or safe shelter. The organization’s mandate might be to offer psychosocial counselling for example, but in a context where the everyday safety is not met, the counselling can be difficult to offer effectively. Gender and age can influence the degree of participation in different types of services, with men generally described as more difficult to reach. People spoke of the lasting impacts of colonial practices on cultural knowledge and ways of living. Institutions are often run by non-Inuit and cultural knowledge is generally understood as an “add-on” to existing services rather than the crux of the everyday functioning of organizations. While communication was deemed good by most Inuit, non-Inuit perceived communication as being very difficult to achieve. They spoke of the language barrier and what they considered to be cultural differences. There was an overwhelming desire for cultural knowledge to be embedded within the organizational fabric by, for example, integrating cultural activities and values in services, ensuring Inuktitut language be the foundation of organizational life, and developing human resource guidelines imbricated within Inuit ways of living and doing within organizations. These practices were understood as necessary for community members to trust and use the existing services. There was a recognition that Inuit workers were required to take on these tasks within organizations, however participants spoke of the challenges of creating safe work environments for Inuit workers.

In a similar line of thought, another contextual reality influencing how organizations function is *geography, and relatedly a shortage of workers*. Indeed, communities in Nunavik are relatively small and remote, which affects the flow of people and goods, as well as the overall development of resources within the community. In general, air travel to and from remote communities can be precarious because smaller aircrafts cannot withstand weather disruptions. The more remote communities also have fewer cargo delivery services and air transportation is more affected by weather disruptions, therefore reducing access to goods from outside of the community. Organizational functioning on a daily basis can be heavily impacted by this lack of material goods as well as low-speed internet. The size of the community also affects the delivery of community services in other ways—larger communities have more infrastructures and workers than smaller ones. Realities such as lack of daycare or having to care for a sick family member means that workers often must miss work. In smaller communities where there is a labour shortage, various organizational strategies are deployed to address this critical issue. In health services, people will take on longer working hours and focus on certain tasks over others that seem less urgent. Social workers may also take on the role of youth protection worker, despite the fact that these are two very distinct jobs with different mandates. Other small organizations within small communities might have to close on certain days or at certain hours if they do not have the necessary staff. Wellness workers are community members who work within health and social services to help community members and health workers communicate. In areas where there is no community wellness worker, it can be difficult for health and social services to connect with community members and communicate in efficient and trusting ways. Housing is also a major issue. With limited housing, there is no living space for outside workers or trainers, thereby limiting opportunities to train the local workforce. These various realities, including the fact that there are limited resources, rapid turn-over, changes in job mandates, and small communities with various needs, led to challenges in team dynamics in work settings. Both within and between, organizational conflicts or tensions arose and workers found various ways of coping with these complex realities on a day-to-day basis.

The facilitators and challenges related to contextual realities that were described by participants are clearly inter-related with some factors including structural conditions (e.g., colonization, geography), and community factors. Many of these contextual realities are impossible for organizations to modify on their own; yet, as will be described below, these contexts are essential to consider as they directly impact the functioning of the organizations and their ability to deliver contextually relevant services.

### Indicators of success associated with the organization

From the discussions held with participants regarding the successes of their organization and the ways in which they have transformed their organization over time, we extracted what could be considered success or “performance indicators” based on their narratives. We grouped indicators of success, as described by participants, into three categories: ability to impact clients, staff, and collective well-being; positive group dynamics and organizational functioning; and accessibility of the service. Below, we describe how each of these indicators is defined by participants.

The *ability to impact client, staff, and collective well-being* was, by far, the most discussed and developed indicator. Participants wanted to see the concrete impacts that their organization was making on those it intended to serve. However, interestingly, participants did not only want to see impacts on a targeted “clientele”. They also considered it important to evaluate the impacts of their organization on the staff itself, and on the community as a whole. For example, services were considered successful when they could offer the community much-needed material resources such as infrastructures, funding opportunities, food, and employment. The ability to offer cultural activities and teachings was another form of success for a community organization. Furthermore, services were considered successful when they could enhance collective knowledge and improve the sense of pride and empowerment within the community. For example, when staff received training in cooking or traditional activities and were then able to offer or share these learnings with the community, the services were seen to support well-being of both a targeted clientele or service, of the staff, and of the community at large. At a clientele level, participants spoke of services that support changes in behaviours among their clients, such as reducing harmful habits. Overall, these impacts were seen as a way of improving community life by increasing community safety, social cohesion, and a sense of well-being. Participants also discussed the importance of staff members feeling pride and valued in their roles as workers. Helping community, providing needed resources, and working with and for the community were elements that supported feelings of accomplishment among workers. For example, Inuit workers in community organizations felt empowered to support their peers through culturally relevant activities, and midwives made it possible for women to give birth close to their families in a culturally safe environment.

In order to achieve these impacts on community, participants described how the organization first had to ensure that they had *positive* *team* *dynamics and organizational functioning*. This indicator captures elements of the workplace environment and culture such as staff working well together, staff enjoying their work, and staff feeling that collaborations with external organizations are efficient and supportive. Good team dynamics include strong communication both within and across organizations. Part of successful team efficiency and dynamics means finding ways to help staff feel comfortable and valued in their work environment. Workplaces with a low turnover were understood as offering better services because longer-term employees became knowledgeable about their work. When staff felt confident and knowledgeable about their roles at work, the team dynamics were described as successful. Having staff obtain certifications was another indicator of success.

Last, participants spoke of a variety of success indicators related to the *accessibility* of what the organization offered to the community. Effective accessibility was generally understood as the ability to develop relationships with people in the community, to be trusted by the community, and to be consistent in this relationship over time. Accessibility was also described in terms of physical location (not having to travel too far to access the service) and in terms of consistency over time (being able to rely on it being open in the future).

### Organizational facilitators and needs to achieving success

To improve the services offered by an organization, participants described concrete actions that could be taken around both the *organizational approach* and the *organizational offer*. As community leaders explained, these offers and approaches should be developed based on the organizational and community vision. However, putting these in place requires certain conditions that will be discussed below.

*Organizational approaches* include all the ways in which staff interact among themselves, with clients and with community. Approaches that are effective in connecting with community build on relationships and trust based on a strength-based orientation where people are each respected for their journeys and personal gifts. Here, a welcoming, open, respectful, and comfortable environment greatly promotes the accessibility of an organization. Focusing on language and communication between the organization and community are other important components of culturally safe and strength-based approaches. These positive approaches require that each staff member be supported in their own healing and wellness, and that group dynamics are heavily prioritized through team-building activities, self-care, and offering possibilities for capacity building through training in fields that are directly connected to the interests and strengths of each worker.

*Organizational offer* refers to the type of program or the type of service that is offered by the organization. For example, teenage girls and young women may prefer swimming at the pool during a swim period reserved exclusively for girls and women. These adaptations also include opening hours, the quality of the infrastructures, and making sure the service is known, with clear mandates being communicated. Adapting to culture was described by participants as perhaps the most important way of ensuring accessibility and therefore success of a service. Culturally grounded services are more trusted by people in the community, and they also help build this trust with non-Inuit workers. This kind of organizational offer requires the cultural competencies of Inuit and the ability for non-Inuit to be aware and encourage community customs and practices. Culturally grounded services also require more Inuit support workers such as interpreters, assistants, and aids. And more importantly, Inuit decision-makers within organizations to ensure the values and cultural ways are engrained within the services.

Given the contextual realities including the limited resources to meet community needs, *inter-organizational collaboration* was described by participants as very important and helpful in organizational success. Many organizations explained that they depend on collaboration because they have overlapping mandates (like health services, social services, and the police). One single service often does not have all the resources needed to deliver programs, services, or activities. For example, summer camps conducted by a community organization cannot be successful if parents are not involved, if the municipal government does not contribute goods, if non-Inuit workers are not guided and supported in culturally safe ways of working, and if the Coop store does not provide discount or free food for the camp. Likewise, a community feast cannot be organized without hunters, cooks, and collaboration from a school or recreational hall. Moreover, collaboration is a way of enhancing the quality of services offered and ultimately for improving the impact of services on communities. Yet inter-organizational collaboration is not an easy feat, especially if organizations must deal with rapid worker turnover and inconsistent scheduling or changes in workers’ mandates.

Finally, the Inuit leaders who participated in the analysis (one of whom is a co-author) explained that in order for organizations, programs, or services to be successful, they had to be allowed and encouraged to move away from more typical approaches that generally use pre-established criteria of efficiency. They felt that a more constructive approach to evaluation would be based on long-term planning around community visions and realities. If organizations had access to long-term funding and stable work conditions for employees within organizations, they would then be able to focus on organizational needs, and on responding to community needs. The Inuit leaders imagined gatherings in each community where all the services within the community would be mapped out. Organizations could assess their organizational needs, offers, and approaches. Community members could develop a vision for the community that would allow each organization to establish how they respond to this long-term vision. Each organization could then establish their action plans to ensure that group dynamics, accessibility, and impact were achieved over time.

### Creating a preliminary framework of indicators and the factors that impact upon organizational success

**Fig. 1:**
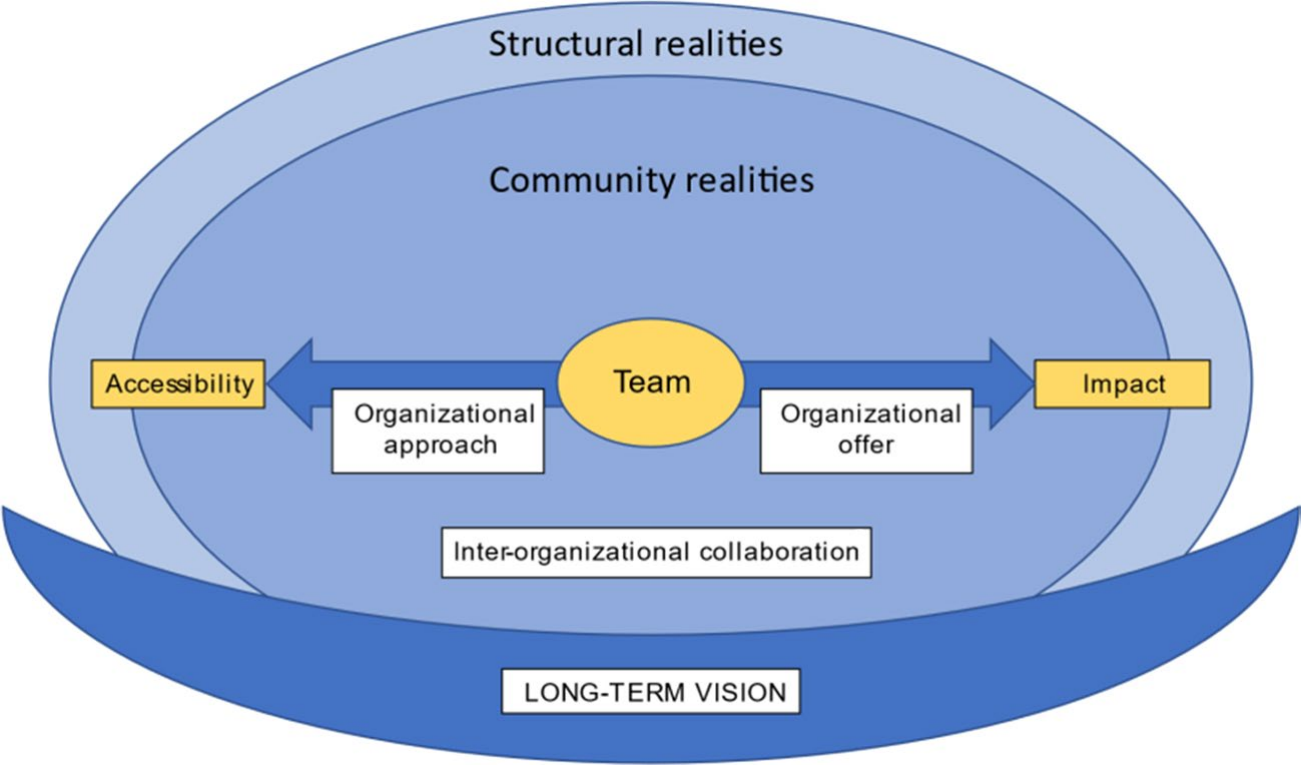
Indicators of success and the factors that influence community organizations

Critical and transformational evaluations allow for constant reflection and action grounded in the contextual realities of an organization, recognizing structural inequalities and dynamics that might impact its functioning. Drawing on our qualitative inquiry, we have developed a culturally and contextually adaptive framework for evaluating organizational services in Inuit communities based on the experiences and perspectives of workers in Nunavik and two local Inuit leaders (see Figure 1). The two large circles are the structural realities and the community realities in which an organization is circumscribed and offer an ecological analysis of the factors that influence organizations. This includes realities such as coloniality, racism, government policies, funding opportunities and expectations, services available in the community and lack of services, availability of human resources and the housing required for these resources, impacts of colonization and coloniality on people’s well-being, and the impacts of trauma on individuals and human interactions. In the middle of the model is the work team and its members (leaders and team members) who are central to the success of the organization, and who are also key resources in each of the communities. Placing them at the centre is a reminder of the importance of focusing on the well-being of team members, their feelings of competence, and their work conditions in order to ensure the retention of staff and the cultural safety of the services that are offered. These are a first set of indicators of success that can be assessed. Once the teamwork and organizational dynamics are fairly stable, then organizational impact and accessibility are two additional types of indicators that can be assessed using a variety of criteria. Achieving success around these large indicators requires certain approaches, the ability to offer a certain form of service, the ability and possibility to work collaboratively with other organizations and the possibility to ground the organizational planning around a long-term organizational, and community vision. This also requires financial stability, and space to try out new approaches, without the fear of losing funding. To illustrate the importance of a long-term vision as described by the leaders, it was integrated into the visual representation at the bottom, like a *Qullik* (traditional seal-oil lamp) holding the fire — all the other components of the model together.

## Discussion

This study aimed to develop culturally and contextually adaptive indicators for evaluating local services and explore the facilitators and challenges that influence these indicators of success. A total of 356 organizations throughout Nunavik were identified and mapped out through interviews with key informants from each of the services. Through these interviews, locally defined and contextually specific visions of success were identified in ways that take into account the structural realities and Inuit culture of Nunavik. These findings begin to establish a set of indicators that depart significantly from traditional performance indicators usually expected by funding organizations.

First and foremost, success was rarely described through quantitative performance indicators, or outputs such as the number of people reached or number of sessions held. Instead, the indicators that were described by participants can better be regrouped around team dynamics, accessibility, and impact. People spoke of being able to offer services like infrastructures, cultural opportunities, job opportunities, moments of gatherings, and feelings of pride for target groups of clientele, for organizational staff members, and for the community more generally. Services are viewed as successful when they offer something that is perceived as a *need* within the community. This makes sense in a context where communities lack many resources. New programs and services allow for enhanced collective opportunities and resources and essentially increase future possibilities for the community. Hence, the mere development of a service that is sustainable is viewed positively.

Much emphasis was also placed on organizational or team dynamics. Participants described the importance of stable staff within the organization and supporting the wellness of each staff member. Experiences of trauma, everyday life challenges can impact individuals and their interactions. Moreover, various reports and articles have highlighted the unequal realities of Inuit and non-Inuit workers within the institutional organizations, as well as the unequal realities between government-funded and community-based organizations (i.e., Plourde-Leveillé & Fraser, 2021; Fraser et al., 2021b, Pitutsimajut Research Team and Sukait Steering Committee, 2020). It is important to offer support and focus on team (Re: model) dynamics, including conflict resolution and identifying people’s skills. But also identifying the systemic realities that create suboptimal, and often times unfair, work conditions within organizations. The staff and team stability that is so essential to a sustainable and effective organization is conditional upon equitable environments where micro-aggressions are not part of daily work. Moreover, past research has described how Indigenous organizations that are decolonizing their services often experience the pressure of community and of institutions expecting that they offer complex services for complex problems, all the meanwhile developing organizational guidelines to decolonize their work environment, all the meanwhile offering support to non-Indigenous people who are in a learning process of their own (Fraser et al., 2021a, 2021b, 2021c). The triple tasking in complex social realities can be overwhelming and requires conditions to retain staff and ensure healthy work dynamics.

When looking at the elements of success related to “impact”, important parallels can be made with findings in other recent publications that document indicators of wellness for Inuit communities (Fletcher et al., 2022; Gagnon-Dion et al., 2021). In this study, participants used several Inuit concepts to describe indicators of success such as having skilled staff and feeling sufficiently trained (*Pigunnatsianiq*) within the organization, feeling useful and busy (*Atuutiqatsianiq*), and feeling that one can make a difference in the lives of individuals, families, and the community at large (*Ilitarijaujutsianiq*). In Fletcher et al.’s (2022) work, people spoke of the importance of feeling that one contributes to one’s community and has a sense of purpose. Being able to create an organizational environment that can offer this sense of personal pride and well-being contributes to community well-being. Indeed, community workers are not only service providers; they are also members of families and communities. Their personal and collective identities are blurred, just as personal impact and collective impact are also integrated. Staff want their clients to be well and supported. This confers a sense pride, competence, and contribution to community, which are elements that are essential for well-being (Fletcher et al., 2022; Fletcher & Dunham, 2008; Gagnon-Dion et al., 2021; Healey et al., 2016; Kral et al., 2011; Kral & Idlout, 2012; Waddell et al., 2017). From the perspectives of clientele, clients want to see staff who are caring and who reach out to them. These actions by staff increase clients’ feelings of psychological and cultural safety in accessing services (Fraser et al., 2021a, 2021b, 2021c). Relationship and reciprocity are fundamental to the functioning of many resources. This finding resonates with authors who have explored the dynamic interplay between individual and collective health among the general population and among Inuit communities (Atkinson et al., 2019; Gagnon-Dion et al., 2021; Roy et al., 2018), and more specifically the relationship between service providers’ well-being, organizational functioning, and client perception of services (Black, 2012; Nienaber & Martins, 2020; Perzynski et al., 2018).

To evaluate a service, one way forward might be to identify goals around team dynamics and then slowly work on the outreach to community. Although team dynamics must constantly be nourished, having a stable work climate and team dynamic will allow the organization to allocate more constructive time and energy to developing services. In this sense, it may be of interest to focus not only on hard-to-reach populations, even if there is a great need and desire, but also to focus on creating services, creating opportunities for training staff, creating opportunities for funding, and creating opportunities to decolonize services. Generating social and collective resources that are made by and for communities is a step towards reducing the challenges that will negatively impact organizational success. Indeed, research highlights how organizational development requires certain social conditions including financial stability, abundance of human resources, and consistent leadership within communities (Laverack, 2001; Martiskainen, 2017; Taylor, 2000).

As the Inuit co-authors remind us, this step-by-step process requires a long-term vision in order to remain motivated. Developing a long-term vision requires long-term funding commitments. This challenge speaks to the ongoing paradoxes experienced by Indigenous communities. Organizational development requires medium- and long-term visions with stable funding and the ability to self-govern in a way that ensures that the vision can be met. If funders choose to adopt criteria that position organizations in a mode of having to prove rather than having the time and possibility to adapt to their realities, then they are continually placed in an impossible position. Organizations cannot be expected to create a vision, develop positive working conditions, and team dynamics and offer consistent services that meet the complex needs of people who have been underserved for decades, all within yearly evaluations and funding renewals.

## Conclusion

The way we evaluate the world (and data) is culturally driven. Yet many evaluation frameworks neglect to take cultural specificity into account. Hood et al. (2015), critical author in program evaluation, has developed a contextually responsive evaluation framework to ensure that the evaluator not take on an essentialist view of culture and omit the structural inequalities. Culture is not a set of traditional activities that are added on to a program, it is the worldview that underlines all actions and decisions. In this research, we worked with Inuit partners to reflect on the organization of information to ensure its relevance. However, we believe that the act of creating themes and developing frameworks is in and of itself a way that is engrained in scientific cultures. The model presented here is informed by culture and context through inductive analysis. Yet we caution readers to avoid any impulse to impose such a model on organizations and communities. It should not be seen as being more “true” than the knowledge of community members and ways of working in or developing services. We offer this model as a flexible tool to start conversations based on the realities of specific communities and services. It will certainly require further validation and adaptation from more people working in different communities and different settings. Moreover, indicators of success are but a mere component of a transformative, critical, justice-oriented evaluation. Funders that support community organizations should ensure that conditions are in place to support these forms of evaluation. Conditions will include funding for human resources to take time to build their evaluation and integrate it within their service offer. In the meantime, we share this model so that it may inform and be used for discussion and reflection. We hope that the operationalization of indicators and the discussions around these indicators can help *Nunavimmiut* reflect on ways in which they can both adapt their existing resources and request that institutional resources adapt to their needs. These adaptations can take place around the indicators that have been described in this article, but mostly around the long-term visions that each community has developed for itself.

## Contributions to knowledge

What does the study add to existing knowledge?

- Further reflection on cultural and contextual adaptation of indicators of success in organizational evaluations.
- Indicators of success that are developed by and for Inuit.

What are the key implications for public health interventions, practice, or policy?

- Public health interventions unfold in communities and organizations, and therefore must be adapted to the realities of those who will be offering and receiving the interventions.
- Decision-makers must rethink ways in which they evaluate and fund organizations, allowing them to become impactful and sustainable in their given geographical and social environments.

## Data Availability

The data belong to the Data Management Committee of Qanuilirpitaa? A process is in place through the Nunavik Regional Board of Health and Social Services to access data.

## Footnotes

1 IQI is an acronym for three Inuktitut terms, *ilusirsusiarniq* (relates to the body), *qanuinngisiarniq* (feeling comfortable, content, and without worries or pain), and *inuuqatigiitsianiq* (harmonious relations among people who share a place) (see Hamel et al., 2020).
